# Hot Climate and Humidity Do Not Interfere with BA.5 Omicron Spreading

**DOI:** 10.1128/spectrum.03294-22

**Published:** 2023-01-12

**Authors:** Maria Grazia Cusi, Chiara Terrosi

**Affiliations:** a Virology Unit, Department of Medical Biotechnologies, University of Siena, Siena, Italy; University of Georgia

**Keywords:** Omicron BA.5, SARS-CoV-2, climate, heat

## LETTER

Many experts had hoped that SARS-CoV-2 would have gone away as soon as the summer weather became warmer and more humid. However, people are wondering why the Omicron BA.5 variant is spreading so much during this summer, when heat extremes have increased on a global scale. Despite the virus appearing to be heat-sensitive (1), the hot summer temperatures had no real effect on it.

Our study tried to understand how the virus responds to heat and humidity. To this aim, we compared the viral titers of the Wuhan and Omicron BA.5 viral strains, according to different exposure times, to the current temperature in Italy, which has a daily average of 32 to 37°C with a moderate (41%) to high (74%) percentage of relative humidity (RH). Heating at 35°C was carried out in an unlidded mini dry hot block with a RH of 41% or in an incubator with a RH of 77%. Virus heating was achieved for 30, 60, and 180 min. At the end of the incubation time, the viruses were titrated as previously described (2). Untreated viruses were left on ice while the heating step was carried out.

It appeared that Omicron BA.5 is not more heat-resistant than the wild type (WT) Wuhan strain, independently of the relative humidity (*P* > 0.05) (Table 1). Although the melting temperature of the Omicron variant spike-receptor binding domains (S-RBDs) showed impaired protein thermal stability, due to mutations in the Omicron S-RBD, it is likely that the BA.5 Omicron sublineage might have a tightly packed 3-RBD-RBD interface in the 3-RBD-down S, such as in BA.2S (1), making it more stable. Docking studies showed that the Omicron variant requires less energy to engage with ACE2, contributing to its higher binding affinity with human ACE2 and determining a more contagious transmission (3).

**TABLE 1:** Viral titers, provided as TCID_50_/mL

Heating time at 41% RH	Wild-type	Omicron 2	BA.5	Heating time at 77% RH	Wild-type	Omicron 2	BA.5
0 min	9.28 × 10^5^	3.4 × 10^6^	4.3 × 10^6^	0 min	9.28 × 10^5^	3.4 × 10^6^	4.3 × 10^6^
30 min	2 × 10^5^	3.4 × 10^6^	3.4 × 10^6^	30 min	9.28 × 10^5^	4.3 × 10^6^	2.28 × 10^6^
60 min	6.3 × 10^5^	3.4 × 10^6^	2 × 10^6^	60 min	3.4 × 10^5^	2 × 10^6^	3.3 × 10^6^
180 min	2 × 10^5^	3.4 × 10^6^	2 × 10^6^	180 min	4.3 × 10^5^	1.12 × 10^6^	1.3 × 10^6^

Therefore, the effect of climate on SARS-CoV-2 survival is low and does not represent a barrier to COVID-19 transmission, whereas other features, such as mobility, have more impact on the diffusion of BA.5 Omicron (4). Using all of the available tools to stop the spread of the virus, including masking, ventilation, and social distancing, is still recommended.
